# Cross-sectional study of attitudes toward online continuing dental education in Libya during the COVID-19 pandemic

**DOI:** 10.1371/journal.pone.0296783

**Published:** 2024-01-10

**Authors:** Ranya F. Elemam, Jamal M. El Swiah, Abduallah O. Durda, Nagwa N. Hegazy

**Affiliations:** 1 Department of Restorative Dental Sciences, Gulf Medical University, Ajman, UAE; 2 Faculty of Dentistry, Conservative Dentistry and Endodontics Department, Misurata University, Misurata, Libya; 3 Alshamis Dental Centre, Tripoli, Libya; 4 Faculty of Medicine, Department of Family Medicine, Menoufia University, Menoufia, Egypt; Shahid Beheshti University of Medical Sciences School of Dentistry, ISLAMIC REPUBLIC OF IRAN

## Abstract

During the COVID-19 pandemic, many educational institutions switched to e-learning educational platforms. This approach was essential but raised challenges, particularly in training practitioners for medical emergencies. This approach not only led to global challenges and a need for rapid adaptation, but also raised inequities across countries, with some facing far more technical challenges than others. In Libya, low investment in education technology and unpredictable internet connectivity limited its integration into schools and universities even before the pandemic. The current study reports feedback from an online continuing dental education (CDE) course for dental practitioners that was developed emergently during the pandemic and aimed to address the challenges posed by Libya’s internet environment. Participants were recruited through social media and received an 8-hour online CDE course consisting of three modules. Participants were invited to complete a pre-course demographic/informational survey on a Google form. After passing all modules, students were prompted to complete a post-course survey consisting of 23, five-point Likert scale questions. Respondents included 43 females (74.1%) and 15 males (25.9%). For ~50% of the cohort (n = 32), this was their first online clinical course. 87.9% of post-course participants rated the course as a positive learning experience, while 90.9% agreed their learning outcomes had been achieved. Most participants (97%) agreed the course instructor explained all concepts clearly. In total, 81.8% agreed that the technology effectively supported their learning. Most agreed that a clear demarcation between each course module existed and that the language and depth of the material were adequate. Some students reported technical difficulties, and 33.3% saw repetitions in the modules. However, all post-course respondents said they would recommend the online course to colleagues. Libyan dental practitioners showed high satisfaction levels towards the e-learning process, course content, instructors’ attitudes, and overall e-learning experience despite the inherent e-learning challenges posed in this country.

## Introduction

The COVID-19 pandemic caused an exceptional disruption of dental education across the globe [1–3] but led to particular challenges in some countries, such as Libya, that did not have established or reliable online infrastructure and availability. In accordance with stay-at-home orders to prevent the spread of the virus, dental schools across the world closed their campuses and moved to online didactic instruction [4–7]. Online teaching platforms were introduced to support graduate dental education and continuing dental education (CDE) for dental practitioners, which included basic science training and theoretical components of clinical dentistry [8,9]. In addition, problem-based learning methods incorporating virtual reality elements were applied to endodontic training at the pre-clinical and clinical levels, improving skill development outside the classroom setting [10]. These knowledge-based courses provided dental practitioners with the latest research and industry advancements, which could enhance their clinical skills and improve patient care [11]. However, the online approach to instruction posed concerns and challenges for Libya’s unreliable internet connection and electrical grid, despite online course participants’ overall openness to the e-learning experience [12,13].

The abrupt transition to e-learning during the COVID-19 pandemic raised concerns for dental teaching and general higher education, not just in Libya but in many regions of the world [9]. Mixed satisfaction with online teaching has been widely observed [6,14–16]. Common problems that impair learner satisfaction include a lack of efficiency with online teaching technology, difficulties understanding the concepts taught, and social isolation resulting in limited development of communication skills [11,17,18]. For example, many students reported that e-learning during the pandemic contributed to poor engagement and impaired the learning process, in part due to inconsistent internet access [19]. Many instructors also felt they lacked adequate technical support for smooth operations despite displaying overall competency in content knowledge [20,21]. Despite these challenges, instructors still largely agreed that technology integration was positive [22]. A survey of international medical students found that frequent internet access issues and electricity outages interfered with their learning and diminished satisfaction with their courses, with many students reporting that online teaching was unfavorable and did not develop their clinical skills or foster productive discussions[23]. Concerns about internet connection also weighed heavily on students’ minds in Libya, despite overall satisfaction with the e-learning experience[12,13]. In addition to technical limitations, many students and instructors have reported a preference for in-person instruction because it allows them to interact with other students and their teachers and forge better bonds[12,24,25]. Likewise, a study of dental students showed that students felt e-learning did not adequately prepare them for examinations or build their practical skills. As a result, many students felt e-learning was only appropriate for theoretical lessons[26].

It has been long established that student satisfaction is tied to grades in learning settings [27]. Recent research has found that factors influencing student satisfaction with in-person courses also influence outcomes for online and blended learning programs [28]. A study examining blended learning programs found that student engagement, course structure, and instructor presence are tied to both learner satisfaction and educational outcomes [29]. Similarly, attitudes to e-learning or blended learning programs are positively correlated with performance on examinations [30]. Learner satisfaction does not just apply to individual courses; program performance has also been associated with academic performance and retention [29]. These associations are widespread and robust, as one study examining over 10,000 students in 10 countries during the COVID-19 pandemic found that satisfaction with the e-learning format was tied to better student performance [31].

Since learner satisfaction is intrinsically tied to outcomes, it is critical to elucidate what factors influence student satisfaction with e-learning courses. Current research has identified several factors that affect student satisfaction with (1) online learning and (2) hybrid models that mix in-person instruction with e-learning. For example, students’ perceptions of e-learning self-efficacy directly correlated with student satisfaction [32]. Similarly, teacher involvement, online learning system quality, and service features were considered the strongest factors influencing student attitudes in an international study [31].

While the above literature has contributed substantially to our general understanding of e-learning, online courses for dental practitioners in Libya were in their infancy prior to COVID, and there is little information about participant satisfaction and what factors support feasibility in regions without reliable internet connections. The current study reports findings from a course survey as a first step evaluating whether e-learning was a viable option for CDE in Libya. Importantly, we identified key factors in e-learning model design that supported and encouraged participant engagement and, therefore, built foundational methodology for improving e-learning outcomes in internet-challenged regions. Specifically, we investigated dental practitioners’ attitudes towards a CDE course during the COVID-19 pandemic in Libya and explored its challenges and successes. Although many individuals in Libya did not make routine cleaning visits during the early days of the pandemic, it was imperative to continue training practitioners for endodontic emergencies [33]. We rapidly designed and implemented a series of online CDE courses for dental interns and practitioners in Libya to support training for this critical care. In the current study, we investigated whether specific adaptations to course infrastructure, such as allowing more time to complete the course and complementary lines of communication between teachers and students, improved course satisfaction and willingness to pursue further online learning.

## Materials and methods

### Study design and participants

The study was performed according to the reporting of observational studies using epidemiology (STROBE) guidelines [34,35]. The study protocol and course were reviewed and approved by the Libyan Society of Endodontology. The recruitment process for our study specifically targeted Libyan practicing dentists. The course announcement was strategically disseminated on social media platforms, including the Libyan Society of Endodontology Facebook page, for a 3-week period. The inclusion criterion was a graduate dental degree; this criterion targeted general dental practitioners, interns, and endodontic specialists. The exclusion criterion was absence of a graduate dental degree; no individuals still in their primary dental training qualified for enrollment in the course or surveys. No inclusion or exclusion criteria were applied regarding the age or gender of recruited dental practitioners; practitioners of any age and gender were welcome to participate. A total of 120 participants showed interest by filling out the pre-registration form, with 91 completing the registration and enrollment process.

### Course design

This course was built, in part, on e-learning principles laid out in a previous study, which implemented a similar online learning approach in an inverted virtual faculty development program[36]. The course was delivered on an online Moodle platform during the COVID-19 pandemic. It consisted of three modules, each with specific learning objectives related to different cognitive aspects of clinical practice in treating endodontic emergencies, and was delivered through videos, quizzes, and discussion. The course was designed to accommodate the challenges and limitations of online teaching in Libya. Specifically, while course content could have been covered in three days, the course was offered over a 10-day period; this flexible schedule accommodated participants’ needs, especially in cases of internet or electricity-related difficulties. It was complemented by a WhatsApp group that engaged all participants, enabling swift communication. Within this group, the two facilitators, both experts in endodontics, were easily accessible to address any queries, provide assistance, and closely track participants who faced challenges with timely submissions or required extra time, particularly those who joined the course late or experienced internet or electricity-related difficulties. Course completion was achieved by scoring 60% or higher on each quiz module. For scores less than 60%, participants were prompted to review the content material, retake the quiz, and resubmit their answers.

While hands-on training is critical in dentistry, the primary contribution of this online continuing education training was for practitioners who already had basic training to be brought up to date on the latest techniques and management that would improve outcomes for endodontic emergencies. Importantly, there was no other mode of training during the pandemic, and this specialty training was particularly critical during a time when medical burdens often fell on individuals who were not fully trained in the area they were treating, due to redeployment [37].

To motivate course completion, participants received certificates for completing the course. A score of 60% or more on each quiz was required to receive the certificate and Continuing Education credit. If a participant scored less than 60%, they were allowed to go back, study the material again, retake the quizzes, and submit answers again. In addition, participants were required to complete the pre-course questionnaire before accessing the first module. For the post-course questionnaire, we were unable to enforce mandatory participation, resulting in some missing responses.

### Data collection and course evaluation

#### Study goals addressed by data collection

The current study was conducted at a time when there is already dense literature addressing certain questions and conclusions about effectiveness of online learning, but it was not intended to achieve the goal of evaluating a full learning model, such as Kirkpatrick’s model [38]. In particular, given the urgency of its implementation, it was not an appropriate platform for a standard research design, which would have delayed life-saving treatment during the pandemic. The goal of our study was not to test learning; rather, the information collected in this study was intended primarily to evaluate attitudes toward e-learning in a region where e-learning faces significant logistical challenges and skepticism of participants about feasibility. Before designing a study to assess learning, it is necessary to first understand if others are willing to use this method and model of learning (reaction, level 1 of Kirkpatrick’s model). If feasibility and willingness to learn this way are lacking, as previous studies of e-learning in internet-limited regions indicated, it would not be worth developing the courses further. The primary goal of the current study was to determine if the adjustments in flexibility and accessibility in the course design used would lead to more positive outcomes. It is not sound methodology to test a model where the methods are uncertain—this study assessed the feasibility of the methods used, not the outcomes.

#### Pre-course survey

Participants were asked to complete a pre-course demographic and informational survey on a Google form and submit their answers before starting the course modules. The pre-course survey was developed internally and consisted of nine closed-ended questions regarding demographics (gender, employment), endodontic emergencies, dental practices during COVID-19, the frequency of attendance of similar previous courses, and knowledge of recommended guidelines for safe dental practice during the pandemic.

#### Post-course survey

After passing all modules, participants were prompted to complete a post-course survey, also developed internally, consisting of 23 five-point Likert scale questions, wherein a score of 1 denoted “strongly disagree,” and 5 represented “strongly agree.” Questions addressed the participants’ satisfaction with the course content, technology, and instructor. A single open-ended question regarding feedback on the course was also included.

#### Survey design

Both the pre-course and the post-course surveys were self-made by the authors to address e-learning needs for endodontic emergency care in Libya during the pandemic. Because this was the first time this particular need had been encountered, the survey was tailored specifically to evaluate the novel adaptations made to the online course. Due to the urgent nature of course implementation, it was not possible to run pilot studies on the surveys. However, we ran reliability statistics on the Likert scale portion of the post-course survey (the majority of this survey) and this yielded a Cronbach’s Alpha internal consistency score of 0.81, surpassing the acceptable thresholds of both 0.70 for early-stage research and 0.80 for applied research [39] In addition, survey questions, both open-ended and Likert format, closely resembled previously published course surveys [40].

### Ethics statements

The study was approved by the Libyan Society of Endodontology. Informed consent was obtained from all participants verbally through online meetings. Written consent was not required as no clinical data were collected. Verbal consent was documented through audio recordings and notes taken by the researcher. The consent process was thoroughly explained to the participants. All were informed of the study’s purpose, procedure, and potential risks and benefits. The participants voluntarily agreed to participate in the study after confirming their understanding of the information provided. Participants’ confidentiality and privacy were maintained throughout the study, and data were collected and analyzed anonymously. The Libyan Society of Endodontology reviewed and approved the study protocol before initiation.

### Statistical analysis

Data were analyzed using SPSS (statistical package for social science) version 26.0 (SPSS Inc., Chicago, IL, USA). Qualitative data were recorded as percentages "n (%)." To evaluate the internal consistency of the post-course survey, we used Fisher’s Exact Test to ask if there was a significant association between learning outcome Likert ratings and three questions relating to attitude toward the online course. Specifically, we looked at interaction between “learning outcome of the course on dental practice” with the following three questions: “preference for running the course online,” “desire to take another course,” and “would recommend this course to other colleagues.” Since three tests were done, we used a Bonferroni correction for multiple comparisons requiring p≤0.05/3 or 0.017 for significance.

## Results

### Study samples

The characteristics of the study cohort are summarized in Table 1. A total of 120 dentists registered for the course, with 91 responses submitted (58: pre-course questionnaire; 33: post-course questionnaire). Pre-course respondents included a significantly higher number of females (n = 43; 74.1%) than males (n = 15; 25.9%). Regarding the experience level of the study samples, 51 (87.9%) were general dental practitioners, 6 were endodontists (10.4%), and 1 participant was an intern (1.7%). Of the participants, 32 (55.2%) were attending an online clinical training course for the first time, 20 (34.5%) stated that they had attended similar courses previously, and 6 (10.3%) stated that they attended similar clinical courses on a regular basis.

**Table 1:** Demographic characteristics of the respondents (pre-course survey).

Variables		Total n = 58
**Gender**	Male Female	15 (25.9%) 43 (74.1%)
**Specialty**	General dental practitioners Endodontist Intern	51 (87.9%) 6 (10.4%) 1 (1.7%)
**Frequency of attending courses on clinical guidelines**	First time Sometimes Regularly	32 (55.2%) 20 (34.5%) 6 (10.3%)

### Pre-course participants

Responses from pre-course participants are shown in Table 2. Some stated prior experience in endodontic emergencies in their practice regularly (n = 14; 24.1%), often (n = 32; 55.2%), or rarely (n = 12; 20.7%). Of the participants, 40 (69%) were aware of primary COVID-19 symptoms, 16 (27.6%) were familiar, and 2 (3.4%) were unaware. A total of 13 (22.4%) participants stated that they were familiar with COVID-19 safety protocols, 40 (51.7%) strictly adhered to recommended COVID-19 guidelines for safety, while 5 (8.6%) were unaware of any COVID-19 safety protocols. Regarding WHO-recommended personal protective equipment (PPE), 22 (37.95%) used PPE regularly, 22 (37.95%) sometimes, and 14 (24.1%) rarely. A total of 17 (29.3%) followed British Endodontic Society guidelines, 38 (65.5%) adhered to American Association of Endodontists guidelines, (2) 3.4% followed general guidelines, and 1 (1.8%) followed no guidelines.

**Table 2:** Pre-course survey responses to practice and COVID-19 questions.

**Dental practice**	Frequency of endodontic emergencies in practice	Very often Often Rare	14 (24.1%) 32 (55.2%) 12 (20.7%)
	Awareness of basic COVID-19 symptoms	Yes totally Somewhat No	40 (69%) 16 (27.6%) 2 (3.4%)
	Awareness of COVID-19 safety protocols	Aware Moderately aware Not so aware	13 (22.4%) 40 (69%) 5 (8.6%)
**Attitudes and practices towards COVID-19 pandemic**	Followed recommended guidelines for safe dental practice during the pandemic	Yes strictly Somewhat No	30 (51.7%) 27 (46.6%) 1 (1.7%)
	Used WHO-recommended personal protective equipment (PPE)	Yes Sometimes No	22 (37.95%) 22 (37.95%) 14 (24.1%)
	Recommendation guidelines adhered to	British Endodontic Society American Association of Endodontists General dental guidelines No guidelines	17 (29.3%) 38 (65.5%) 2 (3.4%) 1 (1.8%)

### Perception of E-learning

A total of 33 respondents provided feedback on perceived benefits of the learning modules. Of these, 16 (87.9%) rated the online course as a positive learning experience, with 18 (90.9%) agreeing that the learning outcomes were clearly met. Moreover, 24 (93.9%) agreed that the course instructor was competent, (87.9%) helpful, (93.9%) encouraged participation, and (97.0%) explained all concepts clearly. Regarding the participant-reported effectiveness of the e-learning module, 14 (81.8%) agreed that the technology could effectively support their learning, with 15 (84.8%) rating the flow of the course as appropriate. Most participants (n = 25, 75.8%) felt equally engaged in all course modules, and 31 (94.0%) agreed that a clear demarcation existed between each module. A total of 32 (97.0%) thought the course language was easy to understand, and 26 (78.8%) believed that the course depth was adequate. A total of 26 participants believed the course content to be of sufficient depth, compared to 22 (66.7%) who stated that the course was too challenging. Only 11 participants (33.3%) thought that the course module showed repetition, with 31 (93.9%) in agreement regarding the relevancy of the session activity and 26 (78.7%) of the effectiveness of the quiz (Table 3). Open-ended feedback was generally positive, although one participant mentioned experiencing technical difficulties with instructional videos.

**Table 3:** Learning outcomes and participants’ attitude towards the course instructor and module content (post-course survey).

Survey Questions	Disagree	Neutral	Agree
**Rated the course as a learning experience**	0 (0%)	4 (12.1%)	29 (87.9%)
**Learning outcomes were clearly met**	0 (0%)	3 (9.1%)	30 (90.9%)
**Course instructor was competent**	0 (0%)	2 (6.1%)	31 (93.9%)
**Course instructor was helpful**	0 (0%)	4 (12.1%)	29 (87.9%)
**Instructor encouraged participation**	0 (0%)	2 (6.1%)	31 (93.9%)
**Instructor explained concepts clearly**	0 (0%)	1 (3.0%)	32 (97.0%)
**Technology effectively supported my learning**	0 (0%)	6 (18.2%)	27 (81.8%)
**Rated the sequence and flow of the course**	0 (0%)	5 (15.2%)	28 (84.8%)
**Felt equally engaged in all course modules**	2 (6%)	6 (18.2%)	25 (75.8%)
**Presence of clear demarcation between each course module**	1 (3%)	1 (3%)	31 (94%)
**Language was easy to understand**	1 (3%)	0	32 (97%)
**Course content was of sufficient depth**	0	7 (21.2%)	26 (78.8%)
**Course content was too challenging**	9 (27.2%)	2 (6.1%)	22 (66.7%)
**Repetitions were present in the modules**	16 (48.5%)	6 (18.2%)	11 (33.3%)
**Activity sessions were relevant**	0	2 (6.1%)	31 (93.9%)
**Quizzes were relevant**	2 (6.1%)	5 (15.2%)	26 (78.7%)

*For purposes of the table here we have combined counts of “agree and strongly agree” ratings and “disagree and strongly disagree” ratings.

### Overall E-learning experience ratings

Of the post-course respondents, a higher number (n = 25; 75.8%) stated a preference for the online course compared to an on-site equivalent (n = 8; 24.2%). A total of 32 (97%) agreed that they would retake the course online compared to on-site, and 1 (3%) was neutral to the course. All post-course survey participants (100%) stated that they would recommend the course to other colleagues (**Table 4**).

**Table 4:** Participant preference for the online course (post-course survey).

Participants’ Attitude	Respondents (n = 33)
**Preference for running the course (online) as opposed to on-site.**	25 (75.8%) Online 8 (24.2%) On-site
**Desire to take another online course.**	0 No 1 (3%) Neutral 32 (97%) Yes
**Would recommend this course to other colleagues.**	33 (100%) Agree 0 (0%) Disagree

### Internal consistency of learning outcome ratings and attitude toward online course

There was a trend toward interaction between Likert ratings of learning outcome and “preference for running the course online” (p = 0.039). There were significant interactions between learning outcome and “desire to take another course” (p = 0.007) and between learning outcome and “would recommend this course to other colleagues” (p = 0.017) (**Table 5**).

**Table 5:** Interaction between feedback “implementation the learning outcome of the course on dental practice on Likert scale” and general attitude of post-course questionnaire respondents (post-course survey).

Q	2 (n = 1)	3 (n = 1)	4 (n = 11)	5 (n = 20)	P-value
**1. If we repeat this course again, do you prefer run this course (online) or (in site)?** **Online** **In site**	0 1 (100%)	1 (100%) 0	6 (54.5%) 5 (45.5%)	18 (90%) 2 (10%)	**0.039** *
**2. Would you like to take another online course?** 3 4 5	1 (100%) 0 0	0 0 1 (100%)	0 5 (45.5%) 6 (54.5%)	0 2 (10%) 18 (90%)	**0.007** **
**3. Would you recommend this course to other students?** 4 5	1 (100%) 0	1 (100%) 0	5 (45.5%) 6 (54.5%)	2 (10%) 18 (90%)	0.017* **

**significant

*trend.

## Discussion

Although dentistry is a practice-based specialty, the incorporation of e-learning into dental education is now widespread. A paradigm shift towards online teaching occurred worldwide following the outbreak of the COVID-19 pandemic, including CDE [41–47]. This transition in learning was a new experience for both educators and learners [48,49]. The current study was designed to assess the feasibility and success of e-learning on CDE in Libya. This study’s findings highlight how the concept of virtual learning was novel amongst Libyan dental practitioners, with the majority reporting it as their first experience of an online course. The satisfaction levels of the included participants were relatively high, and feedback was positive; 100% of respondents in the post-course survey said they would recommend the course to other colleagues and 97% reported a desire to take another online course. The specific course design, targeted to the challenges posed by Libya’s online infrastructure, appeared to contribute to the easy adoption and acceptance of the e-learning courses since most of the participants provided positive feedback for the overall experience.

During the initial COVID-19 period, clinicians were eager for further online courses to boost their knowledge but there were no data on approach or feasibility in regions where internet connectivity was not reliable. The course evaluated in the current study was designed to address these challenges to the extent possible without previously established platforms for doing so. Specifically, the course extended over 10 days instead of 3 days, providing flexibility for all participants to access all educational materials and overcome the frequent internet disconnections. This flexibility was critical, as infrastructural issues like these are tied to decreased learner engagement and satisfaction [23]. Moreover, the presence of the facilitators in the WhatsApp group removed doubt when an activity or test was unclear and increased the attendees’ satisfaction. In this program, a strong majority felt that the course instructor was competent (93.9%), helpful (87.9%), and encouraged active participation (93.9%). This is critical, as instructor engagement is strongly correlated to learner satisfaction in other studies [29,31]. Moreover, the use of asynchronous and synchronous discussion forums was reported by learners in one study as critical for engagement[10]. Future programs should be similarly adaptive to technological issues in order to avoid dissatisfaction with e-learning.

An additional advantage of online learning for this specific region and population was that knowledge could be accessed for low cost, time, and effort [50,51]. A relatively large number of participants taking the online course required long journeys to attend the on-site course, making the course accessible to some who might not have attended CDE otherwise. In previous studies investigating the perspective of the participating dentists and instructors, 63.2% preferred e-learning compared to face-to-face teaching [52]. In the current study 75.8% preferred the online to the in-person format; in this particular case, the accessibility challenges with in-person courses in Libya may have played a role in this strong preference, in addition to the other adaptations we made.

During preparation, there were challenges with poor internet connections, difficulties in communicating remotely, and the inability to identify a suitable e-learning platform. In previous studies, difficulties in concentration during online training and the inability to deliver online tasks or assignments were two major reasons cited for the preference for face-to-face training [53,54]. The absence of a face-to-face connection during e-learning is also thought to contribute to professional isolation [55]. In contrast, responses regarding the e-learning module showed that a plurality of participants (81.8%) thought that the technology implemented in the program facilitated their learning, with no respondents reporting that it did not. However, one participant reported technical issues viewing instructional videos in the open-ended comments section at the end of the survey. This example of technical difficulties impacting the overall learning experience mirrors the overall international experience with e-learning and is a potential point of refinement for this program and others [23,47].

Several previous studies have examined the impact of e-learning on the development of clinical skills and outcomes, compared to traditional learning, in dental workers [56–58]. In these studies, attendees stated a lack of confidence in their ability to handle patients as a result of online learning [53,59]. Conversely, e-learning was shown to encourage general dental practitioners to participate in clinical training, with up to 65% reporting positive benefits [60]. These differences may be due to the different modes of learning between preclinical and clinical years. As an example, a computer-assisted design manufacturing (CAD/CAM) learning software for preclinical prosthodontics exercises received positive feedback when implemented [61]. A variety of factors, including attitude, online teaching expertise, learning environment, and the quality of online content may account for these discrepancies [27,62–68]. In addition, economic, cultural, and political issues can impact learner attitudes and perceptions of their education environment [66–71]. However, the program implemented in this study was largely deemed effective by participants, with the majority (90.9%) feeling that learning objectives were met and no respondents disagreeing that the course was an effective learning experience. Similarly, a high degree of engagement was reported by attendees, with 75.8% agreeing that they felt engaged across all modules.

### Strengths and limitations of this study

While the current study focused on the demands and constraints of learning during the COVID-19 pandemic, the findings in this study provided an opportunity to expedite online learning tool development for the post-COVID-19 era. The pandemic drove a steep learning curve for online teaching methods that will benefit the future of online education. The findings in the current study identified factors leading to the success of online course implementation in infrastructure-, internet- and electricity-challenged regions across the world by showing that extended time for completion and inclusion of discussion groups are key factors influencing (1) willingness to participate in online courses and (2) ability to successfully complete such courses. The challenges addressed by our course design were not themselves related to the pandemic and, thus, are fully applicable to courses beyond the pandemic.

The findings of this study highlight new opportunities for e-learning classes within the Libyan CDE sector. However, some limitations should be noted. (1) The internally developed survey used in this study had to be developed rapidly and in parallel with the emergent demand for the online course. As a result, there was no time to run this specific survey through a pilot stage. However, the questions closely resembled previously published surveys of this nature[40] and the measure of survey internal consistency was shown to be rigorous (Cronbach’s Alpha = 0.81 and significant within-construct consistency). (2) During the delivery of this course, the pre-course questionnaire was obligatory, but the post-course questionnaire was optional, leading to a drop-off in responses. As a result, the sample size was small, with 25 responders lost to follow-up between pre- and post-questionnaires. There is thus a possibility of response bias toward those who felt more engaged in the course. It is noteworthy that this study was completed at a time when many research programs around the world were shut down completely, so it is a strength that this study was completed, even if a certain percentage of participants were lost to follow-up. (3) The large proportion of high-performing individuals in the sample taking the survey could have led to a volunteer bias.

Below, we suggest recommendations for future research practice based on what we learned from the strengths and limitations of the current study.

### Implications of this study for research practice

This study was intended to capture participant reactions to the first online continuing dental education courses in Libya, building the foundation of technical and infrastructural feasibility needed to design a more comprehensive course evaluation. With this logistically feasible platform now established, future studies can refine the course and replicate and extend the survey findings. There are many elements to learning success and the current study honed in on only very focused, specific factors but, importantly, these were factors that were critical to the context of this particular course [72]. Future studies are needed now (1) to evaluate the range of other elements that affect course success, including more direct assessment of learning itself, and (2) to assess the interconnectedness of individual factors in determining this success.

One future research recommendation would be a more comprehensive evaluation of course effectiveness, including direct assessment of participant learning. The current study evaluated only the first level (Reaction) of Kirkpatrick’s model of learning evaluation (Kirkpatrick and Kirkpatrick [38]); future studies can now be planned to assess levels 2 and 3 of this model to evaluate course content learning and implications of such learning. For example, level 2 (Learning) could be assessed more directly using pre- and post-tests for course material, and level 3 (Behavior) could be assessed using surveys about how the course has changed clinical practice methods. Follow-up studies with a control group receiving equivalent face-to-face training would permit comparison of the online versus in-person approach to teaching this material.

Another future research recommendation would be to compare, in a non-emergent situation (i.e., not during a pandemic), the course offering with versus without the adjustments made to promote technical/logistical feasibility; this would allow for more direct testing of the role of these adjustments in improving feasibility. Such a study might, to be fair and ethical, use a cross-over design to be certain all participants have access to the material by the end of the study, although one could not directly compare learning within-subject across conditions in this manner. Another research design might entail comparing the same course methods with two different instructors, since learning has been shown to be strongly dependent on the beliefs and attitude of the instructor [73]. Collection and analysis of survey data regarding the course instructor’s own experience would also be useful in this regard.

Another future research recommendation would be to adjust certain design elements of the current study. For example, in the current study, only the pre-course questionnaire was required to receive the course certificate and continuing education credit. To better motivate participation in the post-course questionnaire, future studies could require both the pre- and post-course questionnaires be completed to receive the course certificate and credit. Studies in larger cohorts would also be beneficial; for this purpose, further increases in motivation, such as monetary incentives, might be included with participation.

Finally, studies at multiple locations are now required to confirm the generalizability of the results to other disciplines, instructors, and participant pools.

## Conclusion

In conclusion, the overall satisfaction of Libyan dental practitioners toward (1) the e-learning process, (2) online course content, and (3) instructor’s attitude and experience was high for the online course evaluated in this study. Easy access to knowledge, the lack of travel to attend the course, flexibility in the schedule, the ability to retake the course, and the presence of facilitators were cited as major advantages of both e-learning generally and the specific design of this course. A lack of experience with online courses and internet instability were recognized as pre-existing challenges to implementing online CDE, particularly in countries such as Libya. The COVID-19 pandemic has emphasized the need for prompt development of adaptive and effective strategies to make learning accessible and facilitate the transition from face-to-face to remote learning for dental, medical, and paramedical professions. Future studies can now build on the strengths and learn from the limitations of the current work.

## Abbreviations

CAD/CAM: Computer-assisted design manufacturing
CDE: Continuous dental education
e-learning: Electronic learning
PPE: Personal protective equipment
SPSS: Statistical package for social sciences

## References

[1] Bastos RA, Carvalho D, Brandao CFS, Bergamasco EC, Sandars J, Cecilio-Fernandes D. Solutions, enablers and barriers to online learning in clinical medical education during the first year of the COVID-19 pandemic: A rapid review. Med Teach. 2022;44(2):187–95. doi: 10.1080/0142159X.2021.1973979 . 34608845

[2] Connolly CA. Response to solutions, enablers and barriers to online learning in clinical medical education during the first year of the COVID-19 pandemic. Med Teach. 2022;44(12):1426–7. doi: 10.1080/0142159X.2022.2042468 . 35188848

[3] O’Doherty D, Dromey M, Lougheed J, Hannigan A, Last J, McGrath D. Barriers and solutions to online learning in medical education—an integrative review. BMC Med Educ. 2018;18(1):130. doi: 10.1186/s12909-018-1240-0 ; PubMed Central PMCID: PMC5992716. 29880045 PMC5992716

[4] Bahanan L, Alsharif M, Samman M. Dental Students’ Perception of Integrating E-learning During COVID-19: A Cross-Sectional Study in a Saudi University. Adv Med Educ Pract. 2022;13:839–47. doi: 10.2147/AMEP.S376069 ; PubMed Central PMCID: PMC9375577. 35971542 PMC9375577

[5] Escobar A, Rojas-Gualdron DF, Velez LF, Santos-Pinto L. Developing diagnostic skills from preclinical dental education: Caries detection and assessment using e-learning assisted practice. J Dent Educ. 2022;86(10):1382–9. doi: 10.1002/jdd.12936 . 35403228

[6] Martinez-Melo K, Bermeo-Escalona JR, Gidi YTME, Cerda-Cristerna BI. A homemade simulation model improves the impact of e-learning for the practical administration of dental anaesthesia. Eur J Dent Educ. 2022. doi: 10.1111/eje.12855 . 36201359

[7] Taramarcaz V, Herren T, Golay E, Regard S, Martin-Achard S, Mach F, et al. A Short Intervention and an Interactive e-Learning Module to Motivate Medical and Dental Students to Enlist as First Responders: Implementation Study. J Med Internet Res. 2022;24(5):e38508. doi: 10.2196/38508 ; PubMed Central PMCID: PMC9161047. 35583927 PMC9161047

[8] Barnes E, Bullock AD, Bailey SE, Cowpe JG, Karaharju-Suvanto T. A review of continuing professional development for dentists in Europe(*). Eur J Dent Educ. 2013;17 Suppl 1:5–17. doi: 10.1111/eje.12045 . 23581734

[9] Haroon Z, Azad AA, Sharif M, Aslam A, Arshad K, Rafiq S. COVID-19 Era: Challenges and Solutions in Dental Education. J Coll Physicians Surg Pak. 2020;30(10):129–31. Epub 2020/10/30. doi: 10.29271/jcpsp.2020.supp2.129 . 33115587

[10] Elemam RF, Dias J, Hegazy NN. A Systematic Review and Proposed Model for Integrating Virtual Reality Simulation Tools with Problem-Based Learning Method in Preclinical and Clinical Endodontics and Restorative Dentistry. Egypt J Hosp Med. 2022;89(1):4298–307. doi: 10.21608/ejhm.2022.256605

[11] Linjawi AI, Agou S. E-learning Readiness among Dental Students and Faculty Members Pre-COVID-19 Pandemic. J Microsc Ultrastruct. 2020;8(4):168–74. doi: 10.4103/JMAU.JMAU_40_20 ; PubMed Central PMCID: PMC7883505. 33623743 PMC7883505

[12] Farkash MI, Aljabour BA, Abdullah AAM, Abdalla MAE. Measuring the Satisfaction Level of the E-Learning Use at LIMU. 2022 International Conference on Engineering & MIS (ICEMIS),; Istanbul, Turkey 2022. p. 1–5.

[13] El-Mansoury A, El-Naas N, Elkawafi M, Kushan A, Arfan S. ONLINE PROBLEM-BASED LEARNING (PBL) DURING CORONAVIRUS PANDEMIC: TRIAL AT THE LIBYAN INTERNATIONAL MEDICAL UNIVERSITY. International Journal of Advanced Research. 2021;9:1036–42. doi: 10.21474/IJAR01/13837

[14] Goh CE, Lim LZ, Muller AM, Wong ML, Gao X. When e-learning takes centre stage amid COVID-19: Dental educators’ perspectives and their future impacts. Eur J Dent Educ. 2022;26(3):506–15. doi: 10.1111/eje.12727 ; PubMed Central PMCID: PMC9011897. 34813667 PMC9011897

[15] Mladenovic R, Matvijenko V, Subaric L, Mladenovic K. Augmented reality as e-learning tool for intraoral examination and dental charting during COVID-19 era. J Dent Educ. 2022;86 Suppl 1(Suppl 1):862–4. doi: 10.1002/jdd.12780 ; PubMed Central PMCID: PMC8657534. 34420217 PMC8657534

[16] Tabatabaei SH, Mirzaiean A, Keshmiri F. Opportunities and threats of e-learning in dental education in viewpoints of faculty members: A Mixed method study. Dent Res J (Isfahan). 2022;19:89. ; PubMed Central PMCID: PMC9680818. 36426275 PMC9680818

[17] Kumar PM, Gottumukkala S, Ramesh KSV, Bharath TS, Penmetsa GS, Kumar CN. Effect of e-learning methods on Dental education: An observational study. J Educ Health Promot. 2020;9:235. doi: 10.4103/jehp.jehp_209_20 ; PubMed Central PMCID: PMC7652085. 33209927 PMC7652085

[18] Takenouchi A, Otani E, Sunaga M, Toyama T, Uehara H, Akiyama K, et al. Development and evaluation of e-learning materials for dental hygiene students in six schools: Using smartphones to learn dental treatment procedures. Int J Dent Hyg. 2020;18(4):413–21. doi: 10.1111/idh.12452 ; PubMed Central PMCID: PMC7689698. 32593228 PMC7689698

[19] Baig QA, Abbas Zaidi SJ, Alam BF. Perceptions of dental faculty and students of E-learning and its application in a public sector Dental College in Karachi, Pakistan. J Pak Med Assoc. 2019;69(9):1320–5. . 31511718

[20] Akram H, Aslam S, Saleem A, Parveen K. The Challenges of Online Teaching in COVID-19 Pandemic: A Case Study of Public Universities in Karachi, Pakistan. J Inf Technol Educ: Res. 2021;20:263–82.

[21] Akram H, Yingxiu Y, Al-Adwan AS, Alkhalifah A. Technology Integration in Higher Education During COVID-19: An Assessment of Online Teaching Competencies Through Technological Pedagogical Content Knowledge Model. Front Psychol. 2021;12:736522. Epub 2021/09/14. doi: 10.3389/fpsyg.2021.736522 ; PubMed Central PMCID: PMC8426343. 34512488 PMC8426343

[22] Akram H, Abdelrady AH, Al-Adwan AS, Ramzan M. Teachers’ Perceptions of Technology Integration in Teaching-Learning Practices: A Systematic Review. Front Psychol. 2022;13:920317. Epub 2022/06/24. doi: 10.3389/fpsyg.2022.920317 ; PubMed Central PMCID: PMC9207921. 35734463 PMC9207921

[23] Aslam S, Akram H, Saleem A, Zhang B. Experiences of international medical students enrolled in Chinese medical institutions towards online teaching during the COVID-19 pandemic. PeerJ. 2021;9:e12061. Epub 2021/09/17. doi: 10.7717/peerj.12061 ; PubMed Central PMCID: PMC8401755. 34527445 PMC8401755

[24] Meulenbroeks R. Suddenly fully online: A case study of a blended university course moving online during the Covid-19 pandemic. Heliyon. 2020;6(12):e05728. Epub 2020/12/29. doi: 10.1016/j.heliyon.2020.e05728 ; PubMed Central PMCID: PMC7749379. 33367131 PMC7749379

[25] Elberkawi EK, Maatuk AM, Elharish SF, Eltajoury WM, editors. Online Learning during the COVID-19 Pandemic: Issues and Challenges. 2021 IEEE 1st International Maghreb Meeting of the Conference on Sciences and Techniques of Automatic Control and Computer Engineering MI-STA; 2021 25–27 May 2021.

[26] Antoniadou M, Rahiotis C, Kakaboura A. Sustainable Distance Online Educational Process for Dental Students during COVID-19 Pandemic. Int J Environ Res Public Health. 2022;19(15). Epub 2022/08/13. doi: 10.3390/ijerph19159470 ; PubMed Central PMCID: PMC9368722. 35954826 PMC9368722

[27] Dyrek N, Wikarek A, Niemiec M, Owczarek AJ, Olszanecka-Glinianowicz M, Kocelak P. The perception of e-learning during the SARS-CoV-2 pandemic by students of medical universities in Poland—a survey-based study. BMC Med Educ. 2022;22(1):529. doi: 10.1186/s12909-022-03600-7 ; PubMed Central PMCID: PMC9263431. 35804369 PMC9263431

[28] Lockman AS, Schirmer BR. Online Instruction in Higher Education: Promising, Research-based, and Evidence-based Practices. Journal of Education and e-Learning Research. 2020;7(2):130–52. doi: 10.20448/journal.509.2020.72.130.152

[29] Gray JA, DiLoreto M. The Effects of Student Engagement, Student Satisfaction, and Perceived Learning in Online Learning Environments. Int J Leadersh. 2016;11(1).

[30] Kintu MJ, Zhu C, Kagambe E. Blended learning effectiveness: the relationship between student characteristics, design features and outcomes. Int J Educ Technol High Educ. 2017;14. doi: 10.1186/s41239-017-0043-4

[31] Kerzic D, Alex JK, Pamela Balbontin Alvarado R, Bezerra DDS, Cheraghi M, Dobrowolska B, et al. Academic student satisfaction and perceived performance in the e-learning environment during the COVID-19 pandemic: Evidence across ten countries. PLoS One. 2021;16(10):e0258807. Epub 2021/10/21. doi: 10.1371/journal.pone.0258807 ; PubMed Central PMCID: PMC8528294. 34669757 PMC8528294

[32] Aldhahi MI, Alqahtani AS, Baattaiah BA, Al-Mohammed HI. Exploring the relationship between students’ learning satisfaction and self-efficacy during the emergency transition to remote learning amid the coronavirus pandemic: A cross-sectional study. Educ Inf Technol (Dordr). 2022;27(1):1323–40. Epub 2021/07/20. doi: 10.1007/s10639-021-10644-7 ; PubMed Central PMCID: PMC8275635. 34276239 PMC8275635

[33] Ather A, Patel B, Ruparel NB, Diogenes A, Hargreaves KM. Coronavirus Disease 19 (COVID-19): Implications for Clinical Dental Care. J Endod. 2020;46(5):584–95. Epub 2020/04/11. doi: 10.1016/j.joen.2020.03.008 ; PubMed Central PMCID: PMC7270628. 32273156 PMC7270628

[34] Skrivankova VW, Richmond RC, Woolf BAR, Yarmolinsky J, Davies NM, Swanson SA, et al. Strengthening the Reporting of Observational Studies in Epidemiology Using Mendelian Randomization: The STROBE-MR Statement. JAMA. 2021;326(16):1614–21. doi: 10.1001/jama.2021.18236 . 34698778

[35] Wiehn E, Ricci C, Alvarez-Perea A, Perkin MR, Jones CJ, Akdis C, et al. Adherence to the Strengthening the Reporting of Observational Studies in Epidemiology (STROBE) checklist in articles published in EAACI Journals: A bibliographic study. Allergy. 2021;76(12):3581–8. doi: 10.1111/all.14951 . 34022062

[36] Hegazy NN, Soliman SS, Ahmed SA, Ahmed MM. An Inverted Virtual Faculty Development Program for Remote Teaching: Pilot for Replication. Egypt J Hosp Med. 2022;86:384–90.

[37] Panda N, Sinyard RD, Henrich N, Cauley CE, Hannenberg AA, Sonnay Y, et al. Redeployment of Health Care Workers in the COVID-19 Pandemic: A Qualitative Study of Health System Leaders’ Strategies. J Patient Saf. 2021;17(4):256–63. Epub 2021/04/03. doi: 10.1097/PTS.0000000000000847 . 33797460

[38] Kirkpatrick DL. Evaluating training programs: the four levels: First edition. San Francisco: Berrett-Koehler; Emeryville, CA: Publishers Group West [distributor], [1994] ©1994; 1994.

[39] Nunnally JC. Psychometric Theory 3E: Tata McGraw-Hill Education; 1994.

[40] Kolcu Mİ B, Öztürkçü Ö SK, Kaki GD. Evaluation of a Distance Education Course Using the 4C-ID Model for Continuing Endodontics Education. J Dent Educ. 2020;84(1):62–71. Epub 2020/01/25. doi: 10.21815/JDE.019.138 . 31977103

[41] Aggarwal S, Wisely CE, Syed M, Siatkowski RM, Challa P. Learning From the 2021 Ophthalmology Match: Virtual Residency Matching During the COVID-19 Pandemic. J Grad Med Educ. 2022;14(6):674–9. doi: 10.4300/JGME-D-22-00186.1 ; PubMed Central PMCID: PMC9765913. 36591419 PMC9765913

[42] Aldosari AM, Alramthi SM, Eid HF. Improving social presence in online higher education: Using live virtual classroom to confront learning challenges during COVID-19 pandemic. Front Psychol. 2022;13:994403. doi: 10.3389/fpsyg.2022.994403 ; PubMed Central PMCID: PMC9714682. 36467142 PMC9714682

[43] Kanagaraj P, Sakthivel R, Christhumary PC, Arulappan J, Matua GA, Subramanian U, et al. Nursing Student’s Satisfaction With Virtual Learning During COVID-19 Pandemic in India. SAGE Open Nurs. 2022;8:23779608221144933. doi: 10.1177/23779608221144933 ; PubMed Central PMCID: PMC9806365. 36601447 PMC9806365

[44] Onggirawan CA, Kho JM, Kartiwa AP, Anderies, Gunawan AAS. Systematic literature review: The adaptation of distance learning process during the COVID-19 pandemic using virtual educational spaces in metaverse. Procedia Comput Sci. 2023;216:274–83. doi: 10.1016/j.procs.2022.12.137 ; PubMed Central PMCID: PMC9829423. 36643176 PMC9829423

[45] Pickren SE, Harriott EM, Huerta NB, Cutting LE. Impact of COVID-19 on Children’s Attention Deficit Hyperactivity Disorder Symptomology, Daily Life, and Problem Behavior During Virtual Learning. Mind Brain Educ. 2022;16(4):277–92. doi: 10.1111/mbe.12337 ; PubMed Central PMCID: PMC9874801 connection with this article. 36712290 PMC9874801

[46] Xie H, Wang L, Pang Z, Chen S, Xu G, Wang S. Application of problem-based learning combined with a virtual simulation training platform in clinical biochemistry teaching during the COVID-19 pandemic. Front Med (Lausanne). 2022;9:985128. doi: 10.3389/fmed.2022.985128 ; PubMed Central PMCID: PMC9644193. 36388919 PMC9644193

[47] Liu X, Zhou J, Chen L, Yang Y, Tan J. Impact of COVID-19 epidemic on live online dental continuing education. European Journal of Dental Education. 2020;24(4):786–9. doi: 10.1111/eje.12569 32648989 PMC7405200

[48] Alsaywid B, Lytras MD, Abuzenada M, Lytra H, Sultan L, Badawoud H, et al. Effectiveness and Preparedness of Institutions’ E-Learning Methods During the COVID-19 Pandemic for Residents’ Medical Training in Saudi Arabia: A Pilot Study. Front Public Health. 2021;9:707833. doi: 10.3389/fpubh.2021.707833 ; PubMed Central PMCID: PMC8435681. 34527651 PMC8435681

[49] Alshammari T, Alseraye S, Alqasim R, Rogowska A, Alrasheed N, Alshammari M. Examining anxiety and stress regarding virtual learning in colleges of health sciences: A cross-sectional study in the era of the COVID-19 pandemic in Saudi Arabia. Saudi Pharm J. 2022;30(3):256–64. doi: 10.1016/j.jsps.2022.01.010 ; PubMed Central PMCID: PMC9051956. 35498216 PMC9051956

[50] Alvarez-Lopez F, Maina MF, Saigi-Rubio F. Use of a Low-Cost Portable 3D Virtual Reality Gesture-Mediated Simulator for Training and Learning Basic Psychomotor Skills in Minimally Invasive Surgery: Development and Content Validity Study. J Med Internet Res. 2020;22(7):e17491. doi: 10.2196/17491 ; PubMed Central PMCID: PMC7388055. 32673217 PMC7388055

[51] Lim AS, Lee SWH. Is Technology Enhanced Learning Cost-effective to Improve Skills?: The Monash Objective Structured Clinical Examination Virtual Experience. Simul Healthc. 2022;17(2):131–5. doi: 10.1097/SIH.0000000000000526 . 33273417

[52] Schlenz MA, Schmidt A, Wostmann B, Kramer N, Schulz-Weidner N. Students’ and lecturers’ perspective on the implementation of online learning in dental education due to SARS-CoV-2 (COVID-19): a cross-sectional study. BMC Med Educ. 2020;20(1):354. doi: 10.1186/s12909-020-02266-3 ; PubMed Central PMCID: PMC7545382. 33036592 PMC7545382

[53] Abbasi MS, Ahmed N, Sajjad B, Alshahrani A, Saeed S, Sarfaraz S, et al. E-Learning perception and satisfaction among health sciences students amid the COVID-19 pandemic. Work. 2020;67(3):549–56. doi: 10.3233/WOR-203308 . 33185620

[54] Ansar F, Ali W, Khattak A, Naveed H, Zeb S. Undergraduate students’ perception and satisfaction regarding online learning system amidst COVID-19 Pandemic in Pakistan. J Ayub Med Coll Abbottabad. 2020;32(Suppl 1)(4):S644–S50. . 33754524

[55] Wang R, Han J, Liu C, Wang L. Relationship between medical students’ perceived instructor role and their approaches to using online learning technologies in a cloud-based virtual classroom. BMC Med Educ. 2022;22(1):560. doi: 10.1186/s12909-022-03604-3 ; PubMed Central PMCID: PMC9297652. 35854299 PMC9297652

[56] Balasubramaniam N, Kujala S, Ayzit D, Kauppinen M, Heponiemi T, Hietapakka L, Kaihlanen A. Designing an E-Learning Application to Facilitate Health Care Professionals’ Cross-Cultural Communication. Stud Health Technol Inform. 2018;247:196–200. . 29677950

[57] Vaona A, Banzi R, Kwag KH, Rigon G, Cereda D, Pecoraro V, et al. E-learning for health professionals. Cochrane Database Syst Rev. 2018;1(1):CD011736. doi: 10.1002/14651858.CD011736.pub2 ; PubMed Central PMCID: PMC6491176 VP: none known. IT: none known. LM: none known. 29355907 PMC6491176

[58] Walsh K. Reflections of health care professionals on e-learning resources for patient safety. Proc (Bayl Univ Med Cent). 2018;31(1):35–6. doi: 10.1080/08998280.2017.1391580 ; PubMed Central PMCID: PMC5903507. 29686549 PMC5903507

[59] Bock A, Kniha K, Goloborodko E, Lemos M, Rittich AB, Mohlhenrich SC, et al. Effectiveness of face-to-face, blended and e-learning in teaching the application of local anaesthesia: a randomised study. BMC Med Educ. 2021;21(1):137. doi: 10.1186/s12909-021-02569-z ; PubMed Central PMCID: PMC7913455. 33639906 PMC7913455

[60] Pahinis K, Stokes CW, Walsh TF, Cannavina G. Evaluating a blended-learning course taught to different groups of learners in a dental school. J Dent Educ. 2007;71(2):269–78. . 17314389

[61] Park CF, Sheinbaum JM, Tamada Y, Chandiramani R, Lian L, Lee C, et al. Dental Students’ Perceptions of Digital Assessment Software for Preclinical Tooth Preparation Exercises. J Dent Educ. 2017;81(5):597–603. doi: 10.21815/JDE.016.015 . 28461637

[62] Arumugam S, Kumar Subbiah N, Jhansi Priya S. A Study on the Performance and Perception of Medical students in Online Spotter versus Traditional Spotter Examinations. J Adv Med Educ Prof. 2022;10(4):246–52. doi: 10.30476/JAMP.2022.94591.1597 ; PubMed Central PMCID: PMC9589066. 36310672 PMC9589066

[63] Bhattarai B, Gupta S, Dahal S, Thapa A, Bhandari P. Perception of Online Lectures among Students of a Medical College in Kathmandu: A Descriptive Cross-sectional Study. JNMA J Nepal Med Assoc. 2021;59(235):234–8. doi: 10.31729/jnma.6276 ; PubMed Central PMCID: PMC8369538. 34506439 PMC8369538

[64] Hulke SM, Wakode SL, Thakare AE, Parashar R, Bharshnakar RN, Joshi A, Vaidya YP. Perception of e-learning in medical students and faculty during COVID time: A study based on a questionnaire-based survey. J Educ Health Promot. 2022;11:139. doi: 10.4103/jehp.jehp_655_21 ; PubMed Central PMCID: PMC9170216. 35677253 PMC9170216

[65] Maqbool S, Farhan M, Abu Safian H, Zulqarnain I, Asif H, Noor Z, et al. Student’s perception of E-learning during COVID-19 pandemic and its positive and negative learning outcomes among medical students: A country-wise study conducted in Pakistan and Iran. Ann Med Surg (Lond). 2022;82:104713. doi: 10.1016/j.amsu.2022.104713 ; PubMed Central PMCID: PMC9494861. 36164641 PMC9494861

[66] Puskulluoglu M, Nowakowski M, Ochenduszko S, Hope D, Cameron H. Medical students’ perception of e-learning approach (MeSPeLA)—a mixed method research. Folia Med Cracov. 2022;62(2):49–70. doi: 10.24425/fmc.2022.141699 . 36256895

[67] Saurabh MK, Patel T, Bhabhor P, Patel P, Kumar S. Students’ Perception on Online Teaching and Learning during COVID-19 Pandemic in Medical Education. Maedica (Bucur). 2021;16(3):439–44. doi: 10.26574/maedica.2021.16.3.439 ; PubMed Central PMCID: PMC8643544. 34925600 PMC8643544

[68] Ziehfreund S, Reifenrath J, Wijnen-Meijer M, Welzel J, Sauter F, Wecker H, et al. Considering medical students’ perception, concerns and needs for e-exam during COVID-19: a promising approach to improve subject specific e-exams. Med Educ Online. 2022;27(1):2114131. doi: 10.1080/10872981.2022.2114131 ; PubMed Central PMCID: PMC9397442. 35993348 PMC9397442

[69] Menon UK, Gopalakrishnan S, CS NU, Ramachandran R, Poornima B, Sasidharan A, et al. Pilot of a questionnaire study regarding perception of undergraduate medical students towards online classes: Process and perspectives. J Family Med Prim Care. 2021;10(5):2016–21. doi: 10.4103/jfmpc.jfmpc_2141_20 ; PubMed Central PMCID: PMC8208202. 34195141 PMC8208202

[70] Plch L. Perception of Technology-Enhanced Learning by Medical Students: an Integrative Review. Med Sci Educ. 2020;30(4):1707–20. doi: 10.1007/s40670-020-01040-w ; PubMed Central PMCID: PMC8368782. 34457833 PMC8368782

[71] Rana R, Kumawat D, Sahay P, Gour N, Patel S, Samanta R, et al. Perception among ophthalmologists about webinars as a method of continued medical education during COVID-19 pandemic. Indian J Ophthalmol. 2021;69(4):951–7. doi: 10.4103/ijo.IJO_3136_20 ; PubMed Central PMCID: PMC8012965. 33727465 PMC8012965

[72] Ahmady S, Khani H. The situational analysis of teaching-learning in clinical education in Iran: a postmodern grounded theory study. BMC Med Educ. 2022;22(1):520. Epub 20220702. doi: 10.1186/s12909-022-03577-3 ; PubMed Central PMCID: PMC9250741. 35780110 PMC9250741

[73] Khani H, Ahmady S, Sabet B, Namaki A, Zandi S, Niakan S. Teaching-learning in clinical education based on epistemological orientations: A multi-method study. PLoS One. 2023;18(11):e0289150. Epub 20231130. doi: 10.1371/journal.pone.0289150 ; PubMed Central PMCID: PMC10688630. 38032997 PMC10688630

